# Multiple recombinants in two dengue virus, serotype-2 isolates from patients from Oaxaca, Mexico

**DOI:** 10.1186/1471-2180-9-260

**Published:** 2009-12-15

**Authors:** Gerardo Perez-Ramirez, Alvaro Diaz-Badillo, Minerva Camacho-Nuez, Alejandro Cisneros, Maria de Lourdes Munoz

**Affiliations:** 1 Department of Genetics and Molecular Biology, Centro de Investigacion y de Estudios Avanzados del Instituto Politecnico Nacional. Av. Instituto Politecnico Nacional 2508, San Pedro Zacatenco, C.P. 07360, Mexico D. F., Mexico; 2 Genomic Sciences Program, Universidad Autonoma de la Ciudad de Mexico, San Lorenzo #290 Col. Del Valle, Mexico DF; 3 Escuela de Medicina Veterinaria y Zootecnia, Universidad Autonoma Benito Juarez de Oaxaca, Oaxaca, Mexico

## Abstract

**Background:**

Dengue (DEN) is a serious cause of mortality and morbidity in the world including Mexico, where the infection is endemic. One of the states with the highest rate of dengue cases is Oaxaca. The cause of DEN is a positive-sense RNA virus, the dengue virus (DENV) that evolves rapidly increasing its variability due to the absence of a repair mechanism that leads to approximately one mutational event per genome replication; which results in enhancement of viral adaptation, including the escape from host immune responses. Additionally, recombination may play a role in driving the evolution of DENV, which may potentially affect virulence and cause host tropism changes. Recombination in DENV has not been described in Mexican strains, neither has been described the relevance in virus evolution in an endemic state such as Oaxaca where the four serotypes of DENV are circulating.

**Results:**

To study whether there are isolates from Oaxaca having recombination, we obtained the sequence of 6 different isolates of DENV-2 Asian/American genotype from the outbreak 2005-6, one clone of the C_(91)_-prM-E-NS1_(2400) _structural genes, and 10 clones of the E gene from the isolate MEX_OAX_1656_05. Evidence of recombination was found by using different methods along with two softwares: RDP3 and GARD. The Oaxaca MEX_OAX_1656_05 and MEX_OAX_1038_05 isolates sequenced in this study were recombinant viruses that incorporate the genome sequence from the Cosmopolitan genotype. Furthermore, the clone of the E gene namely MEX_OAX_165607_05 from this study was also recombinant, incorporating genome sequence from the American genotype.

**Conclusions:**

This is the first report of recombination in DENV-2 in Mexico. Given such a recombinant activity new genomic combinations were produced, this could play a significant role in the DENV evolution and must be considered as a potentially important mechanism generating genetic variation in this virus with serious implications for the vaccines and drugs formulation as occurs for other viruses like poliovirus, influenza and HIV.

## Background

DEN is a serious cause of mortality and morbidity in the tropical and subtropical regions that infects fifty million people every year; approximately 500,000 of them are hospitalized and 5% to 15% of them die, which is a dramatic data [1]. Positive-sense RNA viruses evolve rapidly, [2-4] allowing the virus population to quickly adapt to new environments and escape from host anti-viral responses. One of the principal causes of genetic diversity in DENV is the error-prone replication with RNA-dependent RNA polymerase (RdRp), [5] so that one genomic mutation occurs in nearly every cycle of virus replication. RNA virus, such as DENV populations at a particular region, may also rapidly change due to periodic selective sweeps[6], by the introduction of foreign strains of virus [7-9,2], and due to intra-serotypic recombination [10-14]. However, there has been considerable debate about whether recombination occurs in DENV [15] and the relevance of any recombination to the development of live-attenuated *flavivirus *vaccines [16,17]. Besides, there is a number of reasons for believing that recombination can occur in DENV and this process is being described with increasing frequency in DENV-1 [13,18] and other members of the family *Flaviviridae *[19-22]. The recombination in DENV was reported in the structural genes region and particularly in E gene sequence through the use of the BOOTSCAN, DIVERSE PLOTS, and LARD software [14].

The co-circulation of multiple DENV populations increases the opportunities for a mosquito vector to ingest several variants by feeding on a number of diverse infected hosts, or for a host to be infected by vectors infected with distinct DENV variants. These conditions exist in Mexico, the Caribbean Area and South-East Asia [23]. This is supported by the fact that there are many reports of multiple serotypes of DENV from single hosts [3,23-25]. Furthermore, it is likely that mixed infections with different genotypes of the same serotype may also occur where they co-circulate [26,27].

Oaxaca, Mexico is one of the states where DENV is endemic and serotypes -1, -2 and -3 of DENV are co-circulating [23]. DENV-2 was reported as the serotype with higher frequency compared with DENV-1, -3 or -4. Six partial sequences of the genes encoding proteins: capsid (C), pre-membrane-membrane (prM-M), envelop (E), and non-structural 1 (NS1) represented as C_(91)_-prM-E-NS1_(2400) _from six different isolates of DENV-2 from the Oaxaca outbreaks 2005-2006 were obtained. In addition, the RT-PCR products of C_(91)_-prM-E-NS1_(2400) _and E genes obtained from the MEX_OAX_1656_05 isolate were cloned and sequenced.

MEX_OAX_1656_05 and MEX_OAX_1038_05 isolates displayed recombination in the prM-E and E-NS1 genes and the parental strains were the Asian/American and Cosmopolitan genotypes. In addition, the E gene sequences from the clone 7 (MEX_OAX_1656_05_C07) showed recombination between the nucleotides 906 to 1047 and the parental strains were Asian/American and American genotypes.

## Results

**Figure 1:**
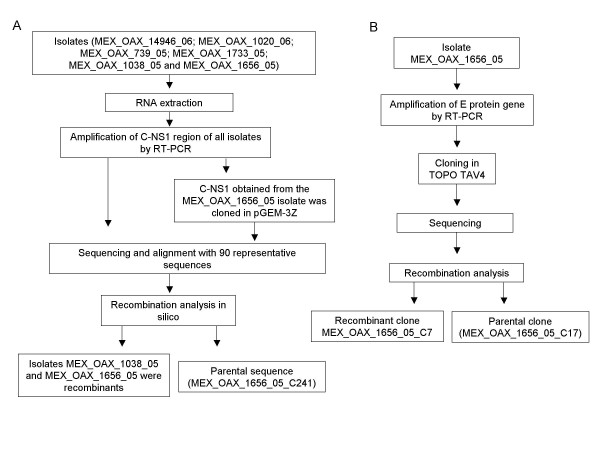
**Experimental strategy**. A) The flow chart shows the experimental strategy that we followed to detect the recombinants in DENV-2 isolates. The C_(91)_-prM-E-NS1_(2400) _region from the MEX_OAX_14946_06; MEX_OAX_1020_06; MEX_OAX_739_05; MEX_OAX_1733_05; MEX_OAX_1038_05 and MEX_OAX_1656_05 isolates and the clone MEX_OAX_1656_05 were amplified and sequenced. All sequences were analyzed with RDP3 and GARD software to detect the recombinants. The analysis *in silico *displayed the recombinants and one parental strain. B) The E protein gene from MEX_OAX_1656_05 was cloned in TOPO TAV4 to detect possible recombinants and/or the parental sequences. One parental sequence was detected in addition to one recombinant.

To determine recombinant sequences in DENV-2, the nucleotide sequences of the partial C_(91)_-prM-E-NS1_(2400) _genome from six isolates and 90 representative sequences of the different genotypes were aligned and analyzed by RDP3 and GARD. In addition, the RT-PCR product of the partial C_(91)_-prM-E-NS1_(2400) _genome from the MEX_OAX_1656_05 isolate was cloned in pGEM-3Z. The sequences of 9 clones were aligned with all of the above sequences (Figure 1A). We also sequenced 10 clones of the E structural gene from the isolate MEX_OAX_1656_05 and aligned with 180 representative sequences containing different genotypes by the programs mentioned above (Figure 1B).

The first task in this phylogenetic analysis was to determine the best model of nucleotide substitution for DENV-2 virus sequence evolution. This assignment was undertaken using the Model Selection test from DataMonkey online server [28,29], which compares 201 models of DNA substitution. Our results demonstrated that the best model was TrN93 [30]. Accordingly, the most complex general time-reversible value was the best fit to the data (relative substitution rates of A↔C = 0.057, A↔G = 1, A↔T = 0.057, C↔G = 0.057, C↔T = 1, and G↔T = 0.057); the Ln likelihood = -4550.59; parameter count = 38; and AIC = 9177.19. Finally, the estimated base composition was A = 0.340, C = 0.278, G = 0.225, and T = 0.157.

**Figure 2:**
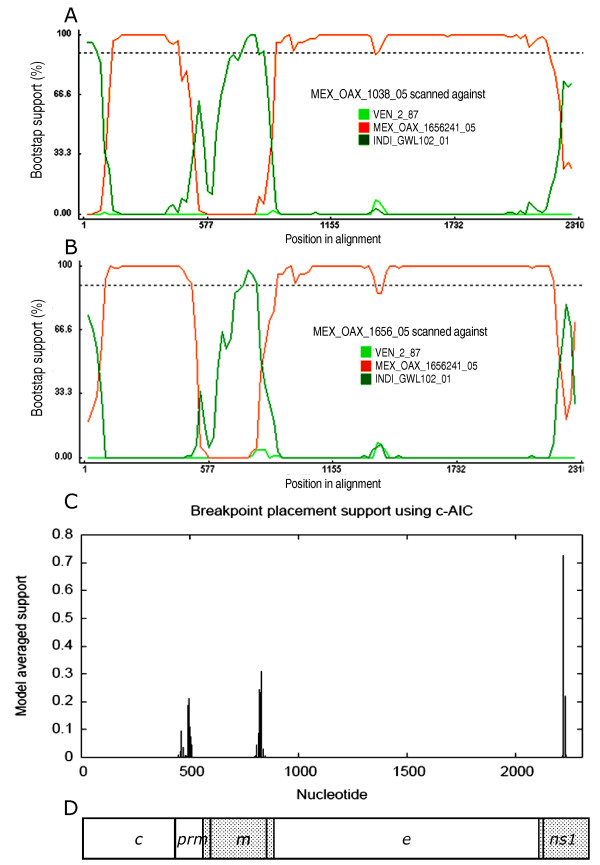
**Recombination plots of structural gene regions from MEX_OAX_1038_05 and MEX_OAX_1656_05 sequences**. A) BOOTSCAN plot analysis of the C_(91)_-prM-E-NS1_(2400) _gene sequences from the MEX_OAX_1038_05 isolate and the parental strains INDI_GWL102_01 and MEX_OAX_1656_05_C241. The first breakpoint is located in the nucleotide 499, the second breakpoint is located in the nucleotide 868 and the third breakpoint is located in the nucleotide 2239; B) BOOTSCAN plot analysis of the C_(91)_-prM-E-NS1_(2400) _gene sequences from the MEX_OAX_1656_05 isolate and the parental strains INDI_GWL102_01 and MEX_OAX_1656_05_C241. The first breakpoint is located in the nucleotide 512; the second breakpoint is located in the nucleotide 826 and the third breakpoint is located in the nucleotide 2239; C) The breakpoint plots of sequences of isolates MEX_OAX_1038_05 and MEX_OAX_1656_05 determined by GARD displayed the first breakpoint in the nucleotide 498, the second breakpoint in the nucleotide 828nt and the third breakpoint in the nucleotide 2226; D) Representation of recombinant regions in the genome of DENV. The nucleotide number is determined for the first nucleotide of our sequence corresponding to the nucleotide 91 starting with the coding region in the C gene.

Our analysis with RDP3 showed that the sequences of isolate MEX_OAX_1038_05 and MEX_OAX_1656_05 present statistical evidence of recombinants for GENECOV (P-Val = 2.467 × 10^-2^), BOOTSCAN (P-Val = 4.289 × 10^-5^), MAXCHI (P-Val = 1.438 × 10^-5^), CHIMERA (P-Val = 3.790 × 10^-3^), SISCAN (P-Val = 1.108 × 10^-9^), and 3SEQ (P-Val = 4.478 × 10^-4^), in two regions (Figure 2): the first breakpoints were located in 499nt and 512nt respectively; the second breakpoints were located in 868nt and 826nt respectively, and the third breakpoint was located in 2239nt in both recombinants (Figure 2A, 2B respectively). In addition, the analysis with GARD confirmed the breakpoints and recombination data for maximum likelihood. This analysis displayed the same site for the three breakpoints in both isolates: the first, second and third breakpoints were located in the nucleotides 498, 828 and 2226, respectively (Figure 2C). The recombinant regions were the intersection of prM-M structural gene to intersection of M-E structural genes and the second recombinant region started in the intersection of E-NS1 genes (Figure 2D). Interestingly, we found that the parental major strain was the non-recombinant clone MEX_OAX_1656_05_ C241 (obtained from the MEX_OAX_1656_05 isolate) and the minor parental strain was the Cosmopolitan genotype strain INDI_GWL_102_01 (accession number DQ448235).

**Figure 3:**
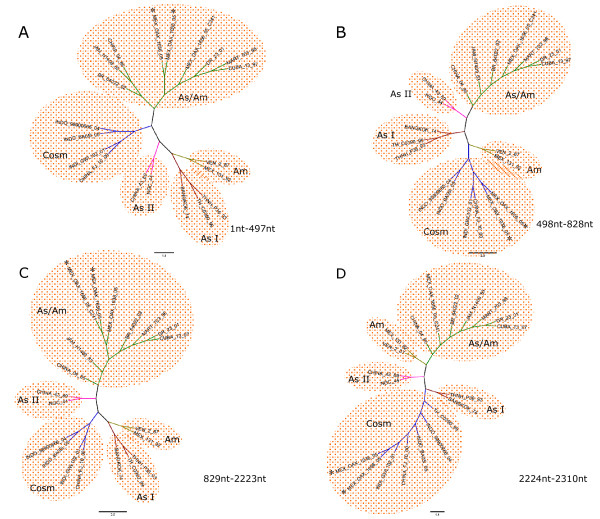
**Phylogenetic trees of MEX_OAX_1038_05 and MEX_OAX_1656_05 based on putative recombination regions**. Maximum Likelihood trees of the putative recombination regions and non-recombination regions of the structural genes C_(91)_-prM-E-NS1_(2400) _of MEX_OAX_1038_05 and MEX_OAX_1656_05 isolates. Nucleotides (nt) 1-497, nt 498-828, nt 829-2222 and 2223-2310 are displayed in A, B, C and D respectively.

The ML tree constructed with our sequence of structural gene C-prM from nucleotide 1-497 from the MEX_OAX_1038_05 and MEX_OAX_1656_05 isolates clustered with the Asian/American genotype (Figure 3A); the analysis of the region from nucleotides 498-828 of the isolates MEX_OAX_1038_05 and MEX_OAX_1656_05 moved to the Cosmopolitan genotype (Figure 3B) and when the region from the nucleotides 828-2222 was analyzed the two strains clustered again with the Asian/American genotype (Figure 3C). Finally, when the region corresponding to nucleotides 2223-2310 was analyzed the isolates clustered with the Cosmopolitan genotype (Figure 3D).

**Figure 4:**
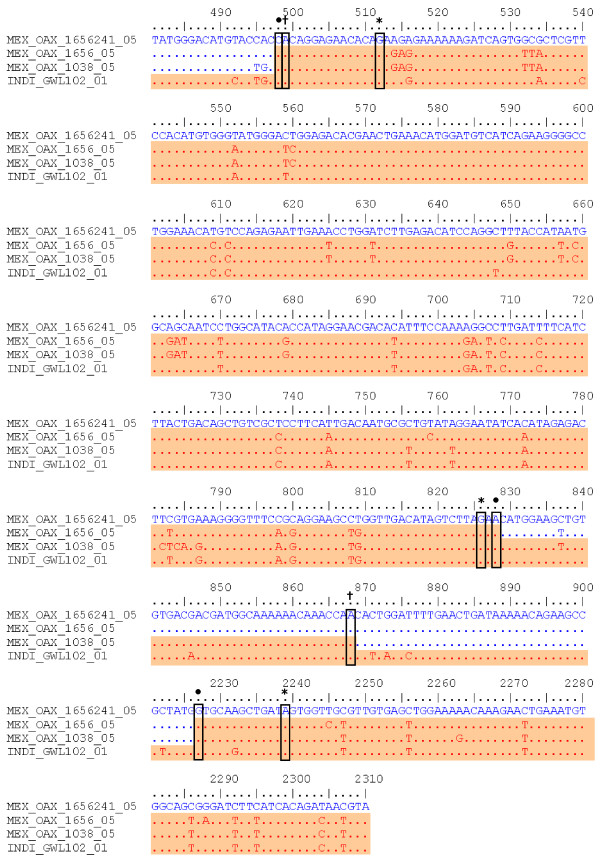
**Nucleotide alignment of C(91)-prM-E-NS1(2400) sequence of MEX_OAX_1038_05 and MEX_OAX_1656_05 putative recombinant isolates with the parental strains**. The number of nucleotide is determined by the position in our sequences of DENV as described in Methods; the location of the breakpoints of MEX_OAX_1038_05 sequence determined for BOOTSCAN is highlighted by (†); the breakpoints of MEX_OAX_1656_05 sequence determined for BOOTSCAN are indicated by (*); the breakpoints of MEX_OAX_1038_05 and MEX_OAX_1656_05 sequences, determined for GARD are labeled by (•). MEX_OAX_1656241_05 clone is the putative mayor parent and INDI_GWI_102_01 is the putative minor parents.

To determine the nucleotides involved in these recombinants, the C_(91)_-prM-E-NS1_(2400) _sequences of the clone MEX_OAX_1656_05_C241, recombinants sequences MEX_OAX_1038_05, MEX_OAX_1656_05 and the Cosmopolitan strain INDI_GWL_102_01 were analyzed. The changes in the recombinant isolates are labeled with a black dot (Figure 4). This analysis showed no evidence of recombination in the recombinant strain MEX_OAX_1656_05.

**Figure 5:**
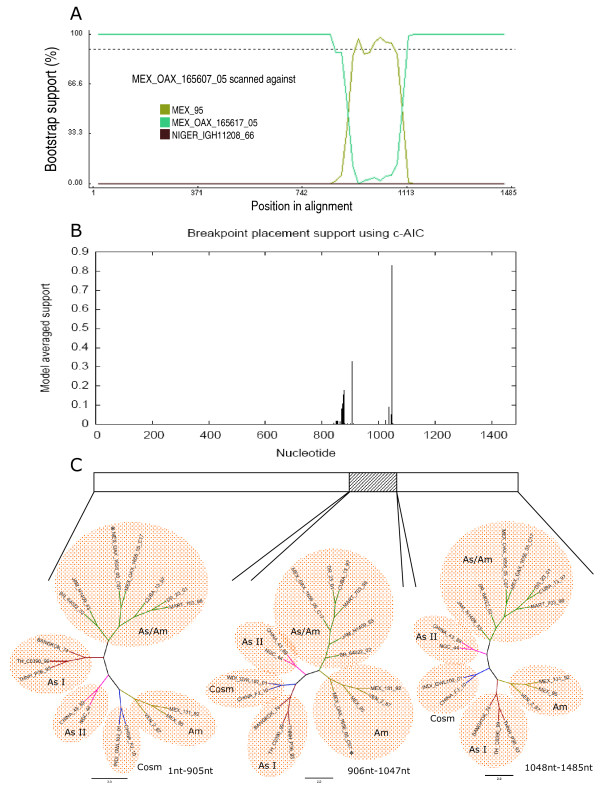
**Recombination plots of clone MEX_OAX_165607_05 of E protein gene**. A) BOOTSCAN plot resulted from the analysis of the clone MEX_OAX_165607_05 sequence with 1000 bootstrap, the putative mayor parent MEX_OAX_165617_05, and the putative minor parent MEX_95; B) Breakpoints plot obtained with GARD algorithm by using the sequences as above; C) Phylogenetic trees (E gene) based on putative recombination and non-recombination regions by maximum likelihood methods.

**Figure 6:**
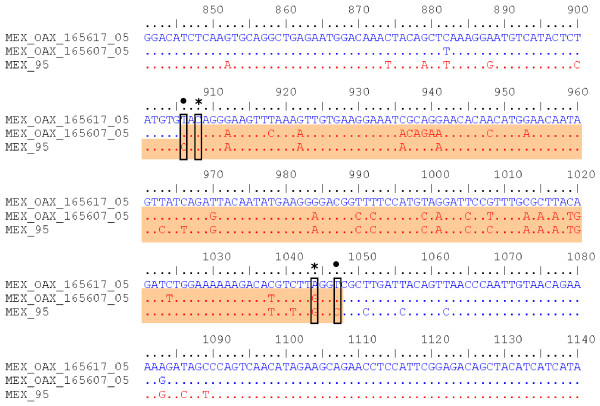
**Alignment of recombinant E protein gene sequence MEX_OAX_165607_05 with parental sequences**. Location of the breakpoints of MEX_OAX_165607_05 sequence determined by BOOTSCAN is highlighted by (*); and the one determined by GARD is labeled by (•). The number of nucleotide is determined by the position in the sequence of E gene.

Like other RNA viruses, DENV undergoes low fidelity replication [31], resulting in virus pools of mutants. Therefore, to determine if one of the parental strains and/or a recombinant sequence is present in these pools, the RT-PCR product of the E protein gene from the recombinant strain, MEX_OAX_1656_05 was cloned and analyzed (Figure 1B). We obtained 10 E protein gene clones that were studied using the RDP3 software and it was determined that the sequence of clone MEX_OAX_1656_05_C07 presents statistical evidence of recombination by GENECOV (P-Val = 7.356 × 10^-7^), BOOTSCAN (P-Val = 1.378 × 10^-5^), MAXCHI (P-Val = 1.764 × 10^-3^), CHIMERA (P-Val = 1.392 × 10^-4^) and 3SEQ (P-Val = 4.478 × 10^-4^). The E protein gene of said clones contains two breakpoints. The first breakpoint was located in the nucleotide 906 of the coding region for protein E; the second breakpoint was located in the nucleotide 1047 of the same gene (Figure 5A, Figure 6). GARD analysis confirmed that this clone is recombinant displaying the first breakpoint in the nucleotide 906 and the second breakpoint in the nucleotide1047 (Figure 5B). The constructed ML trees showed that the MEX_OAX_1656_05_C07 clone clustered in the Asian/American genotype branch when the 1-905 E gene region was examined, and clustered in the American genotype when the E gene region from nucleotide 906 to1047 was analyzed (Figure 5C). Finally, when region 1048-1485 was analyzed, the clone clustered again with the Asian/American strains.

The nucleotides involved in this recombinant are displayed in the alignment of the E gene region sequences of the recombinant MEX_OAX_1656_05_C07 clone, the parental clone MEX_OAX_1656_05_C17 and the strain MEX_95 (Figure 6).

## Discussion

Mutation rate studies indicate that DENV genome averages 1 nucleotide change per cycle of virus replication [32] because of the lack of proofreading activity. Another means to generate genetic changes is through recombination that has been reported in different *Flaviviruses*, including hepatitis C virus (HCV), diarrhea bovine virus (DBV), DENV, Japanese encephalitis virus (JEV), and Saint Louis encephalitis virus (SLEV) [14,16,21]. Recently, some evidences have showed recombination in natural populations of DENV-1 -2, -3 and -4 [13,14,25,33] and the incorporation of this recombination as a probable mechanism contributing to genetic variation in DENV. Furthermore, the circulation of different serotypes and genotypes of DENV in a particular geographical region has been documented [23,34,35], as well as the coexistence of two different serotypes or genotypes in a given mosquito or patient [23,26,27], which makes feasible the recombination in DENV. From the first identification of an intergenotypic DENV recombinant [12], several DENV-1, -2, -3 and -4 recombinant strains have been identified [14]. More importantly, the identification of this recombinant strains demonstrates that DENV is capable of successfully completing all the simultaneous stages of the infection in the same cell: the simultaneous replication of both viral genomes and the template shift by the viral RNA polymerase, while keeping the correct reading frame, encapsidation and release of the recombinant genomes in the process. The products will be subjected to the population processes guiding the maintenance, expansion or disappearance of new variants in the heterogeneous viral population.

All these reports focused on DENV-1 [13,18,27] recombination, and to date, there are a few reports of DEN-2 recombinant strains detected by analysis of protein E sequences [14,25,26]. Besides, protein E gene of clones or C_(91)_-prM-E-NS1_(2400) _region from human serum isolates have not been reported. There is only one single report of putative DENV-2 recombinant clone isolated from mosquitoes in the coding region for protein E [26]. In this report, the isolates MEX_OAX1656_05 and MEX_OAX1038_05 showed recombination within the C_(91)_-prM-E-NS1_(2400) _region. In addition, there was recombination clearly identified within the E protein gene of the clone MEX_OAX1656_05_C7. Furthermore, the parental strains from the recombinants were identified. These results are a strong evidence of the creation of new variants in a heterogeneous viral population. Furthermore, this is the first report of DENV-2 recombination in Mexico.

We detected two isolates containing recombination highly similar to the one obtained from different cities in the state of Oaxaca, which is an evidence of the maintenance and expansion of new variants. These two recombinants in the C_(91)_-prM-E-NS1_(2400) _region contained 3 breakpoints non-previously reported: one in the prM and two in the E protein (Figure 2, 3, 4, 5). We are showing DENV-2 recombination between different genotypes in the isolates and clones analyzed with high frequency of approximately 30% and 10%, respectively. The detection of the DENV recombinants supports a potentially significant role for recombination in the evolution of DENV by creating genetic variation. This result is very important since recombination may shift the virulence of DENV. One could speculate if this shift may increase or diminish the virulence, like has happened in other RNA viruses, such as poliovirus [36,37] influenza virus [38] and the HIV virus [39]. A dramatic example is the loss of the attenuated phenotype of the poliovirus vaccine by recombination, resulting in the generation of new phenotypes that produce the acute paralytic disease. Consequently, recombinants have the potential to generate strains with a higher or lower virulence. To test this issue for DENV recombinants will be necessary to have an animal model to study the virulence of these recombinants.

The two points in our experimental procedure that have been instrumental in obtaining the reported result and to build confidence are: First, we analyzed 6 isolates and one clone in the coding region C_(91)_-prM-E-NS1_(2400) _from Oaxaca and concentrated our efforts in sequencing the E gene of 10 clones from one isolate. These regions were chosen based on its biological relevance and on the location of breakpoints identified in previous reports of recombination in DENV [12,13,26,27,33]; secondly, we minimized the chance of detecting false, artifactual recombination by using long extension times [40] and a proofreading DNA polymerase (Platinum Taq Hi-Fi) [41].

Moreover, the breakpoints tested by RDP3 resulted significant by 7 statistical methods; besides, the GARD software displayed the same breakpoints as the RDP3 software package.

The analysis of 10 clones obtained from the isolate MEX_OAX_1656_05 showed one clone (MEX_OAX_1656_05_C07) containing recombination in the E gene (Figure 5, 6). Interestingly, the parental strains for this recombinant were the Asian/American and the American genotypes. This result is very important because the American genotype has the highest divergence among all the genotypes for DENV-2. Furthermore, this is the first report on recombination between the Asian/American (MEX_OAX_1656_05_C17) and American genotypes (MEX_95), which is supported by the analysis with RDP3 and GARD (Figure 5A-B). This recombinant displays the breakpoints between the nucleotides 906 and 1047. These results suggest that the frequency of recombination in DENV is higher than thought earlier, and the process will remain fundamentally hidden until more studies of clonal diversity to be undertaken. Nevertheless, the precise mechanism underlying the recombination events for DENV is unknown. To understand the mechanism of recombination the development of experimental models for co-infection to generate DENV recombinants is required.

The second breakpoint in the C_(91)_-prM-E-NS1_(2400) _region (nucleotide 868 and 826) for the MEX_OAX_1038_05 and MEX_OAX_1656_05 isolates was different for 40 nucleotides when determined by BOOTSCAN, but it was the same when GARD was used (Figure 4). This was not associated with a sequence that permits the inference of a hot-spot of recombination as previously reported [12,13,26,27] and does not permit the deduction of the mechanism of recombination as has been described for other flavivirus [31][42].

Recombination may be a consequence of circulation of several genotypes at the same time in the same site and probably inside the same cell in the mosquito or human patient; in addition to the high density of different viruses circulating in the geographic area of Oaxaca. This is supported by a previous work that suggests that density of geographical and temporal sampling increases the probability for identifying recombinant sequences [25].

Phylogenetic studies have shown the circulation of the American [43], American/Asian [23], and Cosmopolitan [44] genotypes in Mexico, which makes feasible their recombination and explains the fact of the Cosmopolitan and American genotypes to recombine with the Asian/American genotype spread more broadly. Our results in combination with previous reports [26] on DENV-2 recombination suggest that the different genotypes of DENV-2 are circulating in the virus pool infecting the mosquitoes or the human cells around the world. Until now, it remains unclear whether the frequency of recombination seen in this and previous studies is driving an increasing virulence of DENV strains. However, the recombinant strains of this study were obtained from the outbreak 2005-2006 where the frequency of DHF cases was higher than the DF cases in comparing to previous epidemics [45]. To elucidate the role of recombination in DENV virulence will be necessary to follow the generation of recombinants in outbreaks from other Mexican states.

## Conclusions

It is unclear whether the recombination events took place in a human host or a mosquito vector co-infected by multiple DENV genotypes. In this study, we detected two recombinant isolates of DENV-2 from human hosts namely MEX_OAX_1038_05 and MEX_OAX_1656_05, which identify 3 breakpoints within the prM-E-NS1 genome. Particularly the recombination appeared to have involved two genotypes of DENV-2, the Asian/American clone (MEX_OAX_1656_05_C241) from the same strain and the Cosmopolitan strain (INDI_GWI_102_01).

It is remarkable that parental and recombinant viral sequences of protein E were observed in an isolate from a single patient, particularly when the recombination appeared to have involved two genotypes of DENV-2 (Asian/American and the American) from the same geographic area (Oaxaca, Mexico). This is only the second observation of one parental and recombinant of DENV-2 in a population within a single host [26]. There are two more studies where both parental and recombinant viral genomes were observed in a DENV-1 isolate from a single patient. DENV recombination mechanism will be clarified by undertaking more studies of clonal diversity in both human and mosquito vector in Mexico.

## Methods

### DENV infected cells and virus isolation

*Aedes albopictus *clone C6/36 cells were grown at 28°C. After 18 h of culture, cells (2 × 10^6^/100 mm plate) were infected with 0.2 ml DEN-2 inoculums with an input MOI of 600 PFU/cell and were incubated at 28°C for 10 days.

Viruses were isolated as previously described [46] with a few modifications. After 18 h of culture, C6/36 cells (10^6^/15 ml tube) were infected with 0.01 to 0.1 ml of serum specimen per tube, diluted to 1 ml with medium, and incubated for 2 h at 28°C. After one wash, 3 ml MEM was added and the cells were cultivated for approximately 15 days at 28°C (passage number 1). Cells were observed every day and when a cytopathic effect was apparent from syncytium formation and cellular lysis, the cells were harvested and centrifuged at 3000 rpm for 5 min. The pellet was suspended in 0.6 ml of MEM and stored in aliquots of 0.15 ml at -70°C. The supernatant (approximately 2.5 ml) was stored in 2 aliquots of 1 ml and one of 0.5 ml at -70°C. To obtain passages number two and three, C6/36 cells were incubated with 1 ml of the supernatant obtained from the first or second passage for 2 h at 28°C and the same procedure described above was followed. Serotypes and recombination studies in all samples were determined in the isolates MEX_OAX_14946_06, MEX_OAX_1020_06, MEX_OAX_739_05, MEX_OAX_1733_05, MEX_OAX_1038_05 and MEX_OAX_1656_05 obtained from the third culture-passage. All isolates were obtained by the Health Department from patients with DF, except for the isolate MEX_OAX_14946_06 obtained from a patient with DHF [47].

### RNA extraction

Total RNA was extracted from cell culture supernatant using Trizol^® ^LS (Gibco BRL., Gaithersburg, Md.) according to the manufacturer's recommendations. Ethanol-precipitated RNA was recovered by centrifugation and air-dried. The RNA pellet was suspended in 50 μl water treated with diethylpyrocarbonate (DEPC, Sigma-Aldrich) and used as template for Reverse Transcription with the Polymerase Chain Reaction (RT-PCR).

### Reverse transcription-polymerase chain reaction (RT-PCR)

All assays were performed with the ThermoScript™ RT-PCR System containing Platinum Taq Hi-Fi (Invitrogen, Life Technologies). A mixture of 5 μl of total RNA (0.1-0.5 μg), 50 ng of hexamers/reaction, and DEPC-treated water (in a total volume of 50 μl) was incubated at 65°C for 5 min and chilled on ice. The first extension was carried out at 25°C for 10 min and then at 50°C for 90 min. PCR reaction was carried out by incubation of 20 μM of corresponding sense and antisense PCR primers, 2 μl of the cDNA synthesis reaction and 2.4 mM magnesium sulfate as per manufacture's recommendations. Synthetic oligonucleotide primer pairs were designed based on pairwise of different sequences of DENV-2; to amplify and sequence the partial open reading frame genome region C-prM-E-NS1 from nucleotide 91 (C_91_) to 2400 (NS1_2400_): C(+) CAATATGCTGAAACGCGHG and NS1(-) GTTCTGTCCANGTRTGNAC, and for E gene: primers EPP-F (+) GAATGACAATGCGTTGC and EPP-R (-) TCAGCTCACAACGCAACC.

### Cloning

The RT-PCR product of the partial genome (C_91_-prM-E-NS1_2400_) was restricted with *Kpn1 *and ligated in the pGEM^®^-3Z vector (Promega) following previous protocols [48].

The RT-PCR products of E gene were ligated in the pCR^®^4-TOPO vector included in TOPO^® ^TA Cloning Kit for Sequencing (Invitrogen, Life Technologies) according to the manufacturer's instructions

### Sequencing of PCR products

For sequencing the structural genes or E gene the following primers were used: PrM(+) GGATGTCNKCNGAAGG; PrM(-)CCTTCNGMNGACATCC; E(+) GACAGAGGNTGGGGMAATGG; E(-) CCATTKCCCCANCCTCTGTC; E(-) CNCAAGARGGMGCCAGCC; E(+)GGCTGGCKCCYTCTTGNG. The automated sequencing of purified DNA fragments by spin columns (Qiagen, Chatsworth, Calif.) was performed by the cycle-sequencing dye terminator method. The Big Dye Terminator Cycle Sequencing Ready Reaction Kit (ABIPRISM 100, Applied Biosystems, Foster City, CA) was chosen for sequencing. The sequences obtained were deposited in the GenBank database (AF856321-AF856328; AF856341-AF856350).

### Phylogenetic and Recombination studies

TrN93 substitution model was used to make the phylogenetic analysis since this model showed to be the best to analyze DENV sequences by using "Model Selection" implemented in "DataMonkey" [28,29]

The DENV-2 sequences of partial C_91_-prM-E-NS1_2400 _genome (90) or E gene (180) were aligned using Clustal W [49]; keeping the more representative sequences (17 and 16 respectively) to obtain plots and phylogenies trees to evaluate recombination in our isolates and clones. The accession number of sequences are as follow: VEN_2_87 (AF100465), MEX_131-92(AF100469), THNH_P36_93 (AF022441), TH_CO390_99 (AF100462), BANGKOK_74 (AJ487271), NGC_44 (D00346), CHINA_43_89 (AF204178), CHINA_FJ_10_00 (AF276619), INDI_GWL102_01 (DQ448233), INDO_BA05i_05 (AY858035), INDO_ 98900666_04 (AB189124), BR_64022_02 (AF489932), JAM_N1409_83 (M20558), CHINA_04_85 (AF119661), DR_23_01 (AB122020), MART_703_98 (AF208496), CUBA_13_97 (AY702034), MEX_95 (DQ364562). The aligned sequences were analyzed by Recombinant Detection Program version 3 (RDP3) [50] using default parameters (window of 200nt, step of 20nt, Jin and Nei, 1990 [51] substitution models and 1000 bootstrap) and by the genetic algorithm for recombination detection (GARD) [52,29].

## Authors' contributions

GPR obtained the isolates and clones, carried out the RT-PCR assays using   RNA from passages 3 to sequence the partial C91-prM-E-NS12400 genome and E   gene to develop recombination and phylogenetic analysis. ADB determined   serotype and helped in the phylogenetic analysis. MCN participated in   obtaining the clones of E gene. AC, collected serum samples from patients   from Oaxaca and helped to obtain the isolates and clinical data from Oaxaca,   Mexico. GPR and MLM participated in the writing and discussion of results,   helped to review the manuscript and assisted with the literature validation.   MLM proof-read and assembled the manuscript. All authors participated in the   discussion of results and read and approved the final manuscript.  
